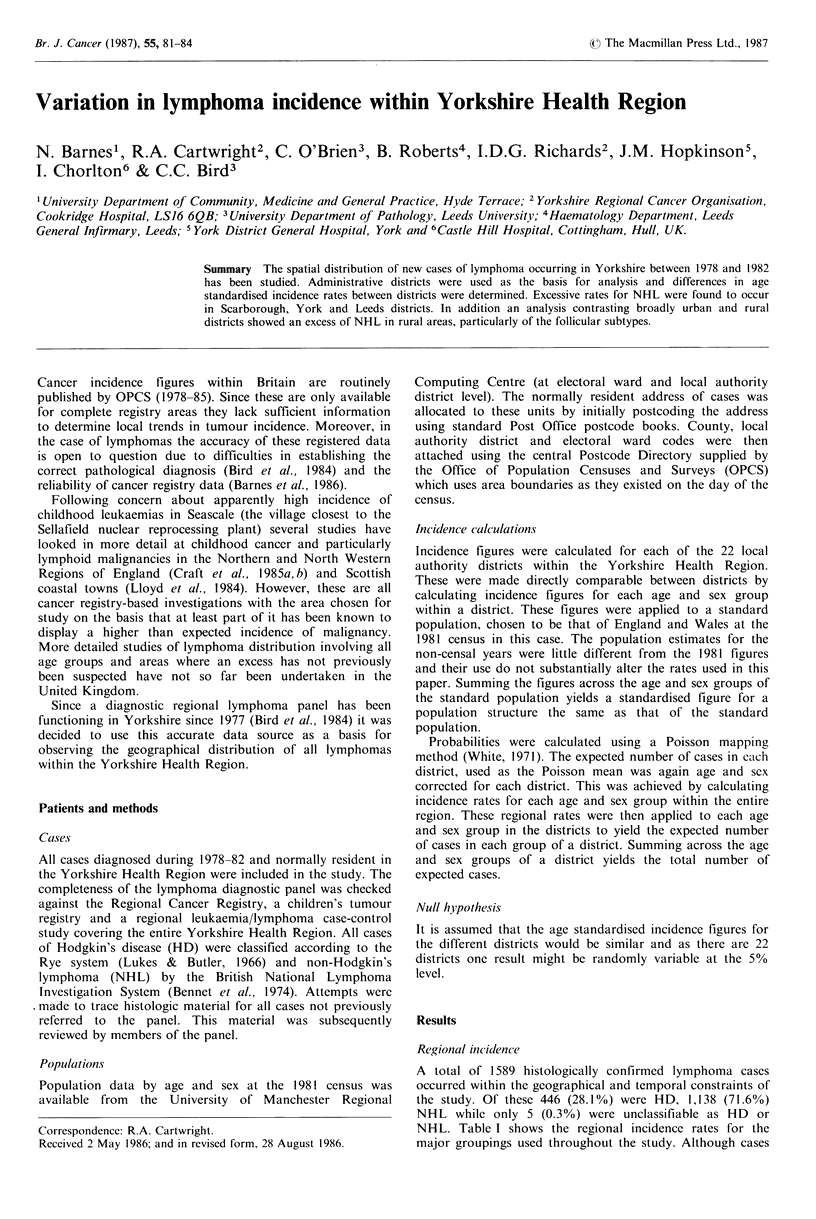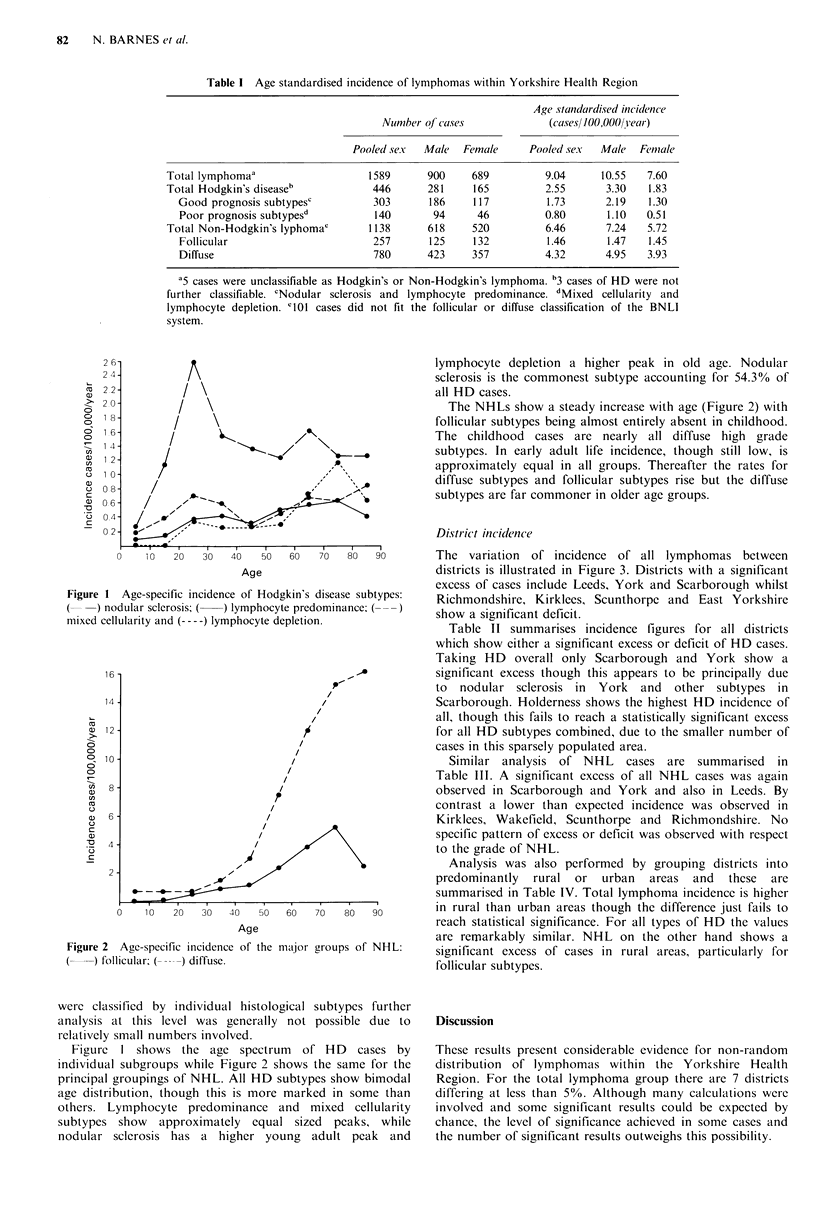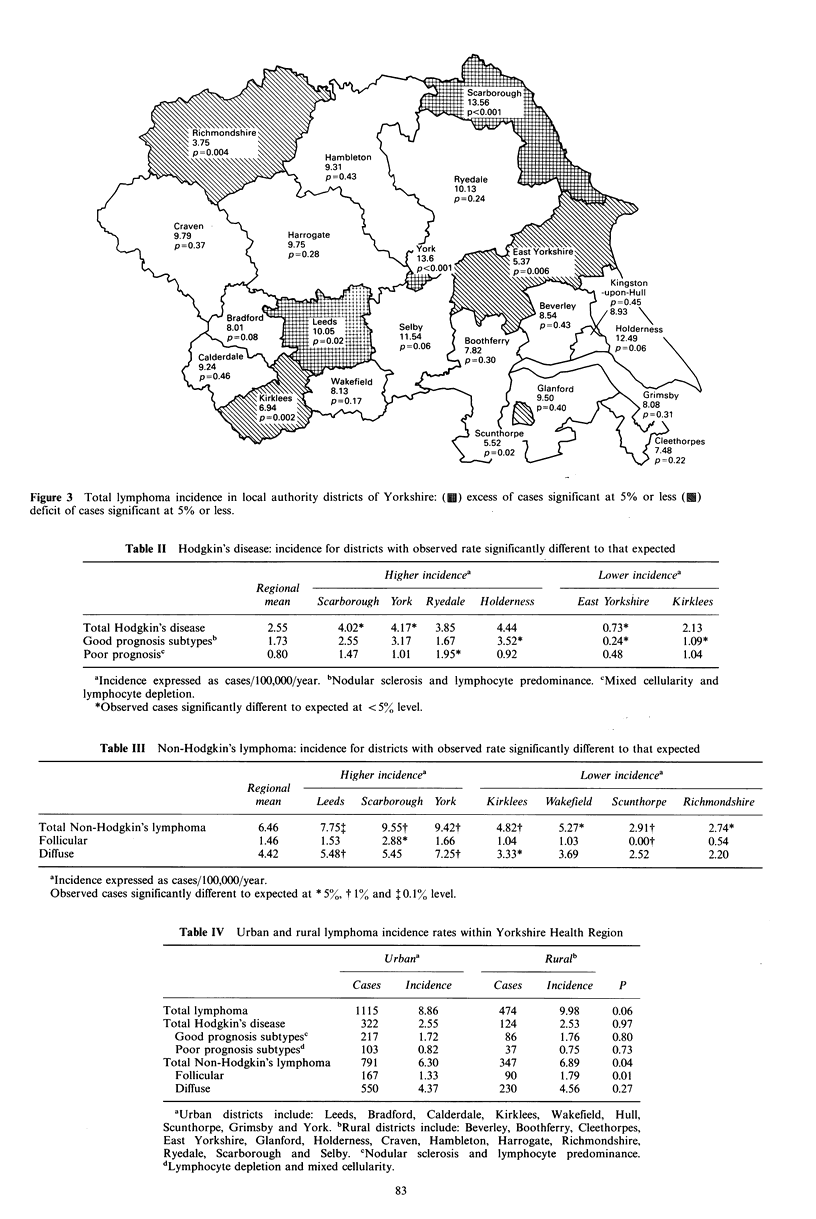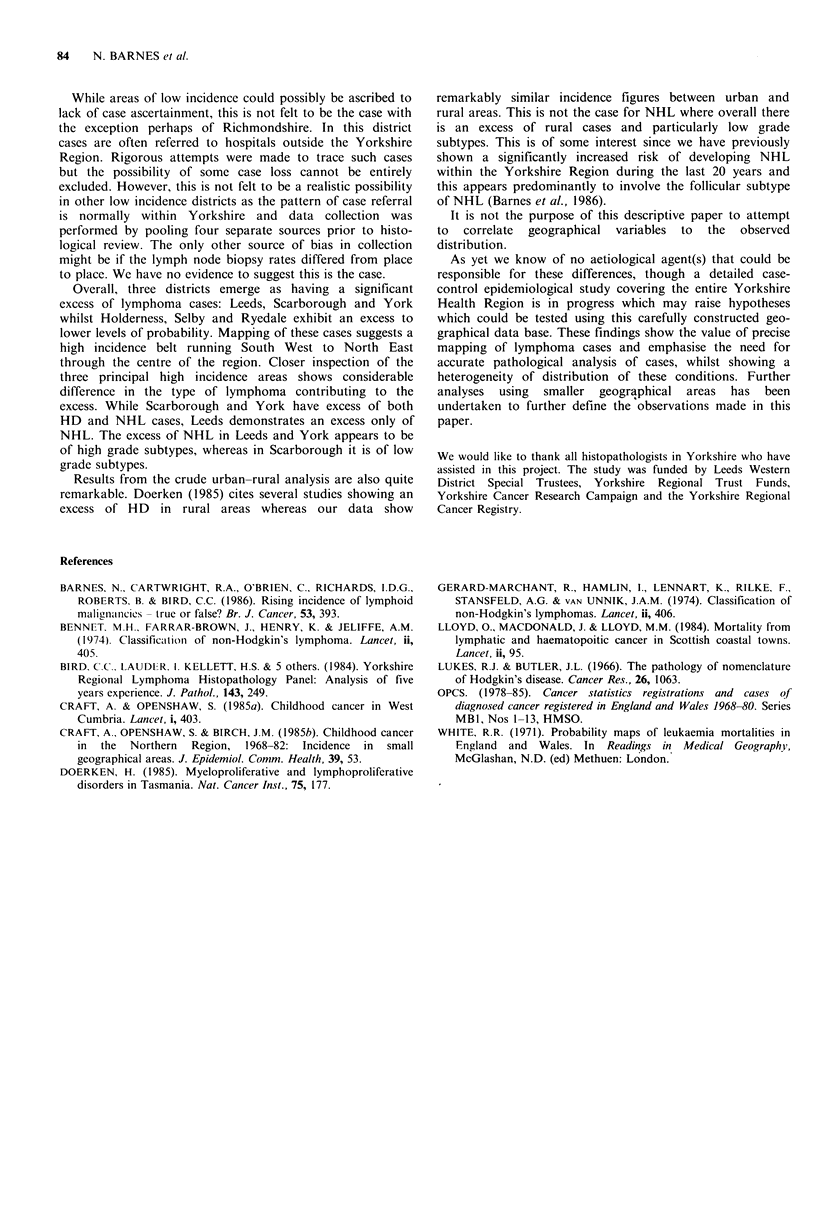# Variation in lymphoma incidence within Yorkshire Health Region.

**DOI:** 10.1038/bjc.1987.17

**Published:** 1987-01

**Authors:** N. Barnes, R. A. Cartwright, C. O'Brien, B. Roberts, I. D. Richards, J. M. Hopkinson, I. Chorlton, C. C. Bird

## Abstract

The spatial distribution of new cases of lymphoma occurring in Yorkshire between 1978 and 1982 has been studied. Administrative districts were used as the basis for analysis and differences in age standardised incidence rates between districts were determined. Excessive rates for NHL were found to occur in Scarborough, York and Leeds districts. In addition an analysis contrasting broadly urban and rural districts showed an excess of NHL in rural areas, particularly of the follicular subtypes.


© The Macmillan Press Ltd., 1987

### Variation in lymphoma incidence within Yorkshire Health Region

N. Barnes[1], R.A. Cartwright[2], C. O'Brien[3], B. Roberts[4], I.D.G. Richards[2], J.M. Hopkinson[5], I. Chorlton[6] & C.C. Bird[3]

[1] *University Department of Community, Medicine and General Practice, Hyde Terrace;* [2] *Yorkshire Regional Cancer Organisation, Cookridge Hospital, LS16 6QB;* [3] *University Department of Pathology, Leeds University;* [4] *Haematology Department, Leeds General Infirmary, Leeds;* [5] *York District General Hospital, York and* [6] *Castle Hill Hospital, Cottingham, Hull, UK.*

**Summary** The spatial distribution of new cases of lymphoma occurring in Yorkshire between 1978 and 1982 has been studied. Administrative districts were used as the basis for analysis and differences in age standardised incidence rates between districts were determined. Excessive rates for NHL were found to occur in Scarborough, York and Leeds districts. In addition an analysis contrasting broadly urban and rural districts showed an excess of NHL in rural areas, particularly of the follicular subtypes.

Cancer incidence figures within Britain are routinely published by OPCS (1978–85). Since these are only available for complete registry areas they lack sufficient information to determine local trends in tumour incidence. Moreover, in the case of lymphomas the accuracy of these registered data is open to question due to difficulties in establishing the correct pathological diagnosis (Bird *et al.*, 1984) and the reliability of cancer registry data (Barnes *et al.*, 1986).

Following concern about apparently high incidence of childhood leukaemias in Seascale (the village closest to the Sellafield nuclear reprocessing plant) several studies have looked in more detail at childhood cancer and particularly lymphoid malignancies in the Northern and North Western Regions of England (Craft *et al.*, 1985*a,b*) and Scottish coastal towns (Lloyd *et al.*, 1984). However, these are all cancer registry-based investigations with the area chosen for study on the basis that at least part of it has been known to display a higher than expected incidence of malignancy. More detailed studies of lymphoma distribution involving all age groups and areas where an excess has not previously been suspected have not so far been undertaken in the United Kingdom.

Since a diagnostic regional lymphoma panel has been functioning in Yorkshire since 1977 (Bird *et al.*, 1984) it was decided to use this accurate data source as a basis for observing the geographical distribution of all lymphomas within the Yorkshire Health Region.

### Patients and methods

### *Cases*

All cases diagnosed during 1978–82 and normally resident in the Yorkshire Health Region were included in the study. The completeness of the lymphoma diagnostic panel was checked against the Regional Cancer Registry, a children's tumour registry and a regional leukaemia/lymphoma case-control study covering the entire Yorkshire Health Region. All cases of Hodgkin's disease (HD) were classified according to the Rye system (Lukes & Butler, 1966) and non-Hodgkin's lymphoma (NHL) by the British National Lymphoma Investigation System (Bennet *et al.*, 1974). Attempts were made to trace histologic material for all cases not previously referred to the panel. This material was subsequently reviewed by members of the panel.

### *Populations*

Population data by age and sex at the 1981 census was available from the University of Manchester Regional Computing Centre (at electoral ward and local authority district level). The normally resident address of cases was allocated to these units by initially postcoding the address using standard Post Office postcode books. County, local authority district and electoral ward codes were then attached using the central Postcode Directory supplied by the Office of Population Censuses and Surveys (OPCS) which uses area boundaries as they existed on the day of the census.

### *Incidence calculations*

Incidence figures were calculated for each of the 22 local authority districts within the Yorkshire Health Region. These were made directly comparable between districts by calculating incidence figures for each age and sex group within a district. These figures were applied to a standard population, chosen to be that of England and Wales at the 1981 census in this case. The population estimates for the non-censal years were little different from the 1981 figures and their use do not substantially alter the rates used in this paper. Summing the figures across the age and sex groups of the standard population yields a standardised figure for a population structure the same as that of the standard population.

Probabilities were calculated using a Poisson mapping method (White, 1971). The expected number of cases in each district, used as the Poisson mean was again age and sex corrected for each district. This was achieved by calculating incidence rates for each age and sex group within the entire region. These regional rates were then applied to each age and sex group in the districts to yield the expected number of cases in each group of a district. Summing across the age and sex groups of a district yields the total number of expected cases.

### *Null hypothesis*

It is assumed that the age standardised incidence figures for the different districts would be similar and as there are 22 districts one result might be randomly variable at the 5% level.

### Results

### *Regional incidence*

A total of 1589 histologically confirmed lymphoma cases occurred within the geographical and temporal constraints of the study. Of these 446 (28.1%) were HD, 1,138 (71.6%) NHL while only 5 (0.3%) were unclassifiable as HD or NHL. Table I shows the regional incidence rates for the major groupings used throughout the study. Although cases

Correspondence: R.A. Cartwright.
Received 2 May 1986; and in revised form, 28 August 1986.

**Table I** Age standardised incidence of lymphomas within Yorkshire Health Region

| | *Number of cases* | | | *Age standardised incidence (cases/100,000/year)* | | |
|---|---|---|---|---|---|---|
| | *Pooled sex* | *Male* | *Female* | *Pooled sex* | *Male* | *Female* |
| Total lymphoma[a] | 1589 | 900 | 689 | 9.04 | 10.55 | 7.60 |
| Total Hodgkin's disease[b] | 446 | 281 | 165 | 2.55 | 3.30 | 1.83 |
| Good prognosis subtypes[c] | 303 | 186 | 117 | 1.73 | 2.19 | 1.30 |
| Poor prognosis subtypes[d] | 140 | 94 | 46 | 0.80 | 1.10 | 0.51 |
| Total Non-Hodgkin's lyphoma[e] | 1138 | 618 | 520 | 6.46 | 7.24 | 5.72 |
| Follicular | 257 | 125 | 132 | 1.46 | 1.47 | 1.45 |
| Diffuse | 780 | 423 | 357 | 4.32 | 4.95 | 3.93 |

[a]5 cases were unclassifiable as Hodgkin's or Non-Hodgkin's lymphoma. [b]3 cases of HD were not further classifiable. [c]Nodular sclerosis and lymphocyte predominance. [d]Mixed cellularity and lymphocyte depletion. [e]101 cases did not fit the follicular or diffuse classification of the BNLI system.

**Figure 1** Age-specific incidence of Hodgkin's disease subtypes: (— —) nodular sclerosis; (——) lymphocyte predominance; (– – –) mixed cellularity and (- - - -) lymphocyte depletion.

**Figure 2** Age-specific incidence of the major groups of NHL: (——) follicular; (- - - -) diffuse.

were classified by individual histological subtypes further analysis at this level was generally not possible due to relatively small numbers involved.

Figure 1 shows the age spectrum of HD cases by individual subgroups while Figure 2 shows the same for the principal groupings of NHL. All HD subtypes show bimodal age distribution, though this is more marked in some than others. Lymphocyte predominance and mixed cellularity subtypes show approximately equal sized peaks, while nodular sclerosis has a higher young adult peak and lymphocyte depletion a higher peak in old age. Nodular sclerosis is the commonest subtype accounting for 54.3% of all HD cases.

The NHLs show a steady increase with age (Figure 2) with follicular subtypes being almost entirely absent in childhood. The childhood cases are nearly all diffuse high grade subtypes. In early adult life incidence, though still low, is approximately equal in all groups. Thereafter the rates for diffuse subtypes and follicular subtypes rise but the diffuse subtypes are far commoner in older age groups.

*District incidence*

The variation of incidence of all lymphomas between districts is illustrated in Figure 3. Districts with a significant excess of cases include Leeds, York and Scarborough whilst Richmondshire, Kirklees, Scunthorpe and East Yorkshire show a significant deficit.

Table II summarises incidence figures for all districts which show either a significant excess or deficit of HD cases. Taking HD overall only Scarborough and York show a significant excess though this appears to be principally due to nodular sclerosis in York and other subtypes in Scarborough. Holderness shows the highest HD incidence of all, though this fails to reach a statistically significant excess for all HD subtypes combined, due to the smaller number of cases in this sparsely populated area.

Similar analysis of NHL cases are summarised in Table III. A significant excess of all NHL cases was again observed in Scarborough and York and also in Leeds. By contrast a lower than expected incidence was observed in Kirklees, Wakefield, Scunthorpe and Richmondshire. No specific pattern of excess or deficit was observed with respect to the grade of NHL.

Analysis was also performed by grouping districts into predominantly rural or urban areas and these are summarised in Table IV. Total lymphoma incidence is higher in rural than urban areas though the difference just fails to reach statistical significance. For all types of HD the values are remarkably similar. NHL on the other hand shows a significant excess of cases in rural areas, particularly for follicular subtypes.

### Discussion

These results present considerable evidence for non-random distribution of lymphomas within the Yorkshire Health Region. For the total lymphoma group there are 7 districts differing at less than 5%. Although many calculations were involved and some significant results could be expected by chance, the level of significance achieved in some cases and the number of significant results outweighs this possibility.

**Figure 3** Total lymphoma incidence in local authority districts of Yorkshire: (▦) excess of cases significant at 5% or less (▧) deficit of cases significant at 5% or less.

**Table II** Hodgkin's disease: incidence for districts with observed rate significantly different to that expected

| | Regional mean | *Higher incidence*[a] | | | | *Lower incidence*[a] | |
|---|---|---|---|---|---|---|---|
| | | *Scarborough* | *York* | *Ryedale* | *Holderness* | *East Yorkshire* | *Kirklees* |
| Total Hodgkin's disease | 2.55 | 4.02* | 4.17* | 3.85 | 4.44 | 0.73* | 2.13 |
| Good prognosis subtypes[b] | 1.73 | 2.55 | 3.17 | 1.67 | 3.52* | 0.24* | 1.09* |
| Poor prognosis[c] | 0.80 | 1.47 | 1.01 | 1.95* | 0.92 | 0.48 | 1.04 |

[a]Incidence expressed as cases/100,000/year. [b]Nodular sclerosis and lymphocyte predominance. [c]Mixed cellularity and lymphocyte depletion.
*Observed cases significantly different to expected at <5% level.

**Table III** Non-Hodgkin's lymphoma: incidence for districts with observed rate significantly different to that expected

| | Regional mean | *Higher incidence*[a] | | | *Lower incidence*[a] | | | |
|---|---|---|---|---|---|---|---|---|
| | | *Leeds* | *Scarborough* | *York* | *Kirklees* | *Wakefield* | *Scunthorpe* | *Richmondshire* |
| Total Non-Hodgkin's lymphoma | 6.46 | 7.75‡ | 9.55† | 9.42† | 4.82† | 5.27* | 2.91† | 2.74* |
| Follicular | 1.46 | 1.53 | 2.88* | 1.66 | 1.04 | 1.03 | 0.00† | 0.54 |
| Diffuse | 4.42 | 5.48† | 5.45 | 7.25† | 3.33* | 3.69 | 2.52 | 2.20 |

[a]Incidence expressed as cases/100,000/year.
Observed cases significantly different to expected at * 5%, † 1% and ‡ 0.1% level.

**Table IV** Urban and rural lymphoma incidence rates within Yorkshire Health Region

| | *Urban*[a] | | *Rural*[b] | | |
|---|---|---|---|---|---|
| | *Cases* | *Incidence* | *Cases* | *Incidence* | *P* |
| Total lymphoma | 1115 | 8.86 | 474 | 9.98 | 0.06 |
| Total Hodgkin's disease | 322 | 2.55 | 124 | 2.53 | 0.97 |
| Good prognosis subtypes[c] | 217 | 1.72 | 86 | 1.76 | 0.80 |
| Poor prognosis subtypes[d] | 103 | 0.82 | 37 | 0.75 | 0.73 |
| Total Non-Hodgkin's lymphoma | 791 | 6.30 | 347 | 6.89 | 0.04 |
| Follicular | 167 | 1.33 | 90 | 1.79 | 0.01 |
| Diffuse | 550 | 4.37 | 230 | 4.56 | 0.27 |

[a]Urban districts include: Leeds, Bradford, Calderdale, Kirklees, Wakefield, Hull, Scunthorpe, Grimsby and York. [b]Rural districts include: Beverley, Boothferry, Cleethorpes, East Yorkshire, Glanford, Holderness, Craven, Hambleton, Harrogate, Richmondshire, Ryedale, Scarborough and Selby. [c]Nodular sclerosis and lymphocyte predominance. [d]Lymphocyte depletion and mixed cellularity.

While areas of low incidence could possibly be ascribed to lack of case ascertainment, this is not felt to be the case with the exception perhaps of Richmondshire. In this district cases are often referred to hospitals outside the Yorkshire Region. Rigorous attempts were made to trace such cases but the possibility of some case loss cannot be entirely excluded. However, this is not felt to be a realistic possibility in other low incidence districts as the pattern of case referral is normally within Yorkshire and data collection was performed by pooling four separate sources prior to histological review. The only other source of bias in collection might be if the lymph node biopsy rates differed from place to place. We have no evidence to suggest this is the case.

Overall, three districts emerge as having a significant excess of lymphoma cases: Leeds, Scarborough and York whilst Holderness, Selby and Ryedale exhibit an excess to lower levels of probability. Mapping of these cases suggests a high incidence belt running South West to North East through the centre of the region. Closer inspection of the three principal high incidence areas shows considerable difference in the type of lymphoma contributing to the excess. While Scarborough and York have excess of both HD and NHL cases, Leeds demonstrates an excess only of NHL. The excess of NHL in Leeds and York appears to be of high grade subtypes, whereas in Scarborough it is of low grade subtypes.

Results from the crude urban–rural analysis are also quite remarkable. Doerken (1985) cites several studies showing an excess of HD in rural areas whereas our data show remarkably similar incidence figures between urban and rural areas. This is not the case for NHL where overall there is an excess of rural cases and particularly low grade subtypes. This is of some interest since we have previously shown a significantly increased risk of developing NHL within the Yorkshire Region during the last 20 years and this appears predominantly to involve the follicular subtype of NHL (Barnes et al., 1986).

It is not the purpose of this descriptive paper to attempt to correlate geographical variables to the observed distribution.

As yet we know of no aetiological agent(s) that could be responsible for these differences, though a detailed case-control epidemiological study covering the entire Yorkshire Health Region is in progress which may raise hypotheses which could be tested using this carefully constructed geographical data base. These findings show the value of precise mapping of lymphoma cases and emphasise the need for accurate pathological analysis of cases, whilst showing a heterogeneity of distribution of these conditions. Further analyses using smaller geographical areas has been undertaken to further define the observations made in this paper.

We would like to thank all histopathologists in Yorkshire who have assisted in this project. The study was funded by Leeds Western District Special Trustees, Yorkshire Regional Trust Funds, Yorkshire Cancer Research Campaign and the Yorkshire Regional Cancer Registry.

### References

BARNES, N., CARTWRIGHT, R.A., O'BRIEN, C., RICHARDS, I.D.G., ROBERTS, B. & BIRD, C.C. (1986). Rising incidence of lymphoid malignancies – true or false? *Br. J. Cancer*, **53**, 393.

BENNET, M.H., FARRAR-BROWN, J., HENRY, K. & JELIFFE, A.M. (1974). Classification of non-Hodgkin's lymphoma. *Lancet*, **ii**, 405.

BIRD, C.C., LAUDER, I. KELLETT, H.S. & 5 others. (1984). Yorkshire Regional Lymphoma Histopathology Panel: Analysis of five years experience. *J. Pathol.*, **143**, 249.

CRAFT, A. & OPENSHAW, S. (1985*a*). Childhood cancer in West Cumbria. *Lancet*, **i**, 403.

CRAFT, A., OPENSHAW, S. & BIRCH, J.M. (1985*b*). Childhood cancer in the Northern Region, 1968–82: Incidence in small geographical areas. *J. Epidemiol. Comm. Health*, **39**, 53.

DOERKEN, H. (1985). Myeloproliferative and lymphoproliferative disorders in Tasmania. *Nat. Cancer Inst.*, **75**, 177.

GERARD-MARCHANT, R., HAMLIN, I., LENNART, K., RILKE, F., STANSFELD, A.G. & VAN UNNIK, J.A.M. (1974). Classification of non-Hodgkin's lymphomas. *Lancet*, **ii**, 406.

LLOYD, O., MACDONALD, J. & LLOYD, M.M. (1984). Mortality from lymphatic and haematopoitic cancer in Scottish coastal towns. *Lancet*, **ii**, 95.

LUKES, R.J. & BUTLER, J.L. (1966). The pathology of nomenclature of Hodgkin's disease. *Cancer Res.*, **26**, 1063.

OPCS. (1978–85). *Cancer statistics registrations and cases of diagnosed cancer registered in England and Wales 1968–80*. Series MB1, Nos 1–13, HMSO.

WHITE, R.R. (1971). Probability maps of leukaemia mortalities in England and Wales. In *Readings in Medical Geography*, McGlashan, N.D. (ed) Methuen: London.